# Factors associated with hematological adverse reactions of drugs authorized via the centralized procedure

**DOI:** 10.1038/s41598-024-59710-3

**Published:** 2024-04-20

**Authors:** Ivana Stević, Slobodan M. Janković, Andrijana Milošević Georgiev, Valentina Marinković, Dragana Lakić

**Affiliations:** 1 Faculty of Pharmacy, University of Belgrade, Belgrade, Serbia; 2 Faculty of Medical Sciences, University of Kragujevac, Kragujevac, Serbia

**Keywords:** Hematological adverse drug reaction, Regulatory factors, Centralized procedure, Adverse effects, Drug safety, Drug regulation, Risk factors, Drug regulation, Epidemiology

## Abstract

Serious hematological adverse drug reactions (HADRs) may lead to or prolong hospitalization and even cause death. The aim of this study was to determine the regulatory factors associated with HADRs caused by drugs that were authorized up to July 2023 by the European Medicines Agency (EMA) and to evaluate the frequency of HADRs. Using a cross-sectional approach, the type and frequency of HADRs were collected from the Summaries of Product Characteristics of Drugs Authorized by the EMA and analyzed within proprietary, nonproprietary, and biosimilar/biological frameworks. Multivariate statistical analysis was used to investigate the associations of generic status, biosimilar status, conditional approval, exceptional circumstances, accelerated assessment, orphan drug status, years on the market, administration route, and inclusion on the Essential Medicines List (EML) with HADRs. In total, 54.78% of proprietary drugs were associated with HADRs at any frequency, while anemia, leucopenia, and thrombocytopenia were observed in approximately 36% of the patients. The predictors of any HADR, anemia, and thrombocytopenia of any frequency are generic status, biosimilar status, and inclusion on the EML, while the only protective factor is the administration route. Biosimilars and their originator biologicals have similar frequencies of HADRs; the only exception is somatropin. Knowledge of the regulatory factors associated with HADRs could help clinicians address monitoring issues when new drugs are introduced for the treatment of patients.

## Introduction

An adverse drug reaction (ADR) is defined as “a response to a medicinal product which is noxious and unintended”; when considering ADRs, a causal relationship to a drug is at least a reasonable possibility^1^. If hematological adverse drug reactions (HADRs) are not a consequence of the main mechanism of action of a drug (A-type ADRs), they are unexpected ADRs (i.e., B-type ADRs)^2^. However, regardless of the mechanism of action, HADRs are usually severe, leading to hospitalization or prolonged hospitalization. A single cell line (e.g., anemia), multiple cell lines, or all bone marrow cell lines (e.g., pancytopenia) could be affected, leading to varying severity in terms of the patient’s condition, prolonged hospitalization, and potentially death. Although the prevalence of HADRs is expected to increase with the use of chemotherapeutics—which directly block the multiplication of cells with a short lifecycle (e.g., the prevalence of anemia varies between 30 and 85%)—HADRs associated with nonchemotherapeutics are different, as they affect bone marrow cell lines and blood cells themselves through a variety of mechanisms, which have not been sufficiently studied^3–7^.

It is estimated that ADRs account for 0.9–7.9% of hospitalizations and are the fifth most common cause of death^8,9^. One study showed that 9.01% (4/44) of ADR-related hospital admissions were due to hematological disorders, while another study reported a rate of 26.5%; however, there are no precise data in the literature on the overall frequency of HADRs^8,10^. The true prevalence of ADRs to nonchemotherapeutics can only be established after the drug is granted marketing authorization (MA) and is used for many years. As a rule, ADRs are not reported during clinical trials in the preregistration phase due to the very low incidence of adverse effects (AEs) within clinical development programs^11,12^. As a drug is on the market for a longer period of time, postmarketing surveillance is performed, including but not limited to postauthorization safety studies (PASSs), spontaneous reports, or monitoring real-world data; furthermore, the drug’s Summary of Product Characteristics (SmPC) is updated with new data on all ADRs, including HADRs^11,13^. Therefore, as a rule, it is more risky to use drugs that are relatively new on the market in clinical practice. Drugs authorized via centralized procedures by the European Medicines Agency (EMA) are, in most cases, innovative drugs, orphan drugs, biosimilars, drugs with conditional approval, or other drugs that have been on the market for a short amount of time^14^. To help clinicians grasp the risks of HADRs when using some of the drugs authorized by the EMA, it would be useful to classify all these drugs by the degree of risk of HADRs.

Therefore, the present study aimed to evaluate the frequency of HADRs among drugs authorized through a centralized procedure and to determine the regulatory factors associated with HADRs.

## Methods

The study used a cross-sectional design. The units of observation were drugs authorized for human use by the centralized procedure in the European Union (EU). Three datasets were created: (1) brand (trade)-name drugs, (2) international nonproprietary name (INN) drugs, and (3) biosimilars and their originator biologicals (hereafter referred to as biologicals). These three datasets were considered separately because ADRs are mostly reported for INN drugs, while for biologicals and biosimilars, ADR reporting also demands trade names^11^. Considering that most drugs are prescribed by trade name in clinical practice, we performed an analysis at this level, including all authorized MAs for all drugs. Initially, the EMA table of all European Public Assessment Reports (EPARs) on all drugs authorized via centralized procedures were downloaded, with a cutoff date of 30 June 2023^15^. This table provides regulatory data on drug category, trade/brand name, therapeutic area, INN, active substance, MA number, authorization status, ATC code, whether a product is additionally monitored, whether it is generic or biosimilar, whether it has conditional approval or not, whether it is authorized in exceptional circumstances via accelerated assessment, whether it is an orphan drug, MA date, MA holder, pharmacotherapeutic group, and condition/indication.

**Figure 1:**
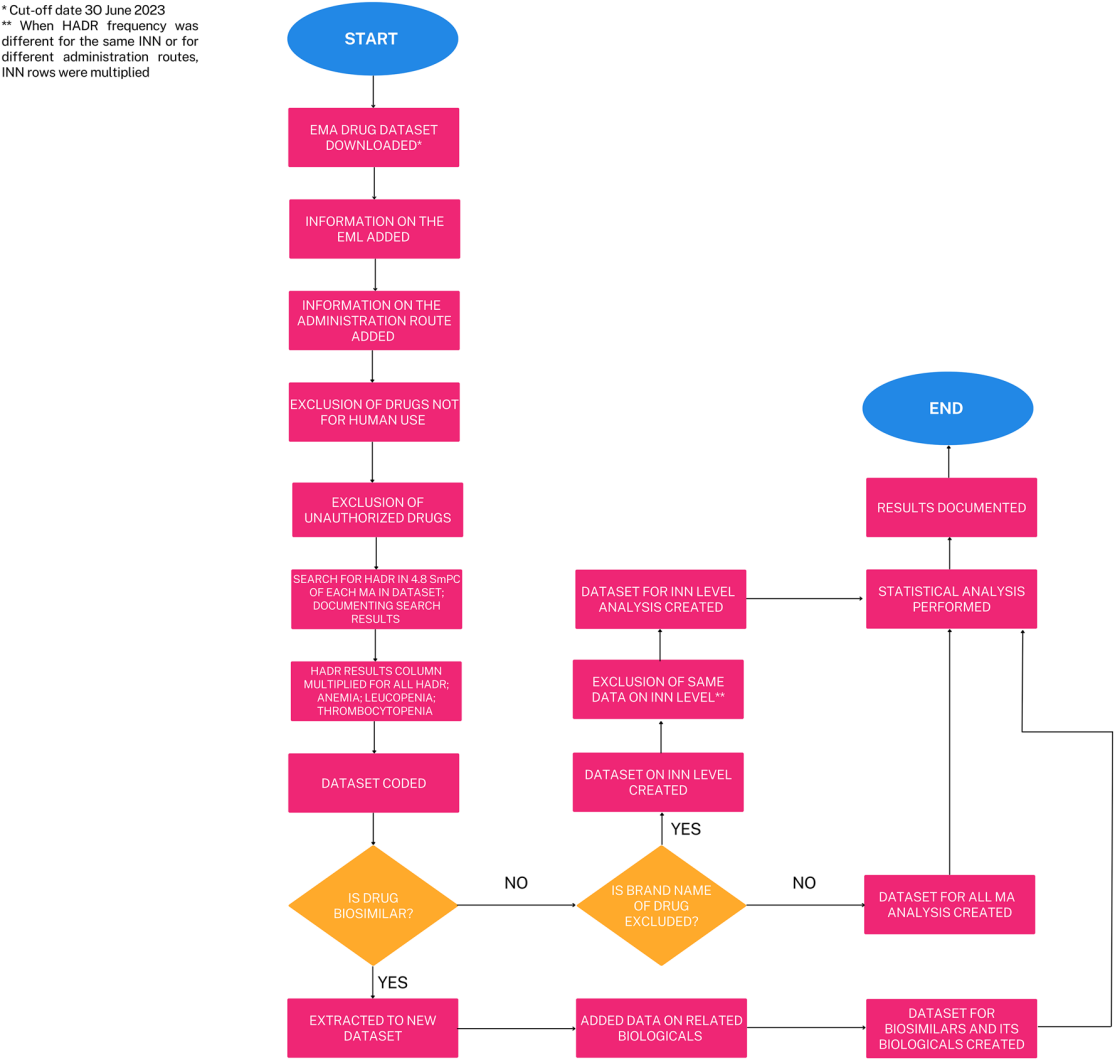
Workflow diagram. EMA, European Medicines Agency; EML, World Health Organization Essential Medicines List; HADR, Hematological adverse drug reaction; INN, International nonproprietary name; MA, Marketing Authorization.

Additionally, in our datasets, we added information on the administration route and whether drugs were on the World Health Organization (WHO) Essential Medicines List (EML)^16,17^. The detailed workflow is provided in Fig. 1. For all included drugs, the HADR data were taken from part 4.8 of the SmPC, considering not only the HADR type but also its frequency.

The incidence of any HADRs, anemia, leucopenia, and thrombocytopenia were considered dependent variables or outcomes of this study. The independent variables for the purpose of this study were additional monitoring, generic status, biosimilar status, conditional approval, exceptional circumstances, accelerated assessment, orphan drug, years on the market (calculated from authorization date until dataset cutoff date), route of administration, and WHO EML.

The data were initially analyzed descriptively using rates and percentages. Then, multivariate binary logistic regression was used to reveal independent variables associated with the study outcomes. The regression models were built using the backward conditional deletion method, and the quality of the final models was verified by the Hosmer and Lemeshow test, Cox & Snell R^2^, and Nagelkerke’s R^2^. The TREE procedure creates a tree-based model. It classifies cases into groups or predicts values of a dependent variable based on values of predictor variables. The procedure provides validation tools for exploratory and confirmatory classification analysis. Significant predictors identified by logistic regression were then used to create decision tree models that classify drugs according to the risk for HADR by creating decision rules.

The results were considered statistically significant if the probability of the null hypothesis was 0.05 or less. The statistical analysis was conducted using IBM SPSS v.29^18^.

## Results

The initial number of MAs in the EMA dataset was 2008. After the exclusion of veterinary drugs (n = 282) and unauthorized (withdrawn, refused) drugs (n = 377), the number of valid MAs for human use included in the statistical analysis was 1349, with 883 unique INNs. When the HADR frequency differed for the same INN (e.g., for different trade names or administration routes), both values were included. The average time from MA approval until study onset for drugs authorized by the EMA was 9.91 ± 6.97 years (0.07–27.93, median 6.96), while for biosimilars and their biologicals, it was 6.01 and 19.08 years, respectively.

In the following sections, we provide detailed results for MA, INN, biosimilars, and their biologicals.

### Analysis of brand-name drugs

Almost the same number of drugs are administered enterally (47.52%) or parenterally (47.59%), while the number of locally administered drugs is much lower (3.19%). One-quarter of all MAs were under additional monitoring, with HADR occurring at any frequency in 47.63% of cases, and at least 30% of cases were anemia, leucopenia, or thrombocytopenia. One-fifth of the drugs were either generic or biosimilar, while one-tenth of the centrally authorized drugs were intended to treat rare diseases. Almost 30% of all MAs were on the WHO EML. Approximately 3% of all MAs were authorized under conditional approval, exceptional circumstances, or through an accelerated procedure.

More than half of the drugs (54.78%) were associated with HADRs, while anemia, thrombocytopenia, and leucopenia were detected in 40.47%, 41.14%, and 36.17% of cases, respectively. One-fifth of the drugs were associated with a very common frequency of any HADR (20.61%), and 15% of the MAs were associated with a very common frequency of anemia or leucopenia. One-fifth of the drugs on the EML were associated with HADRs; a total of 7.19% of the drugs were associated with a very common frequency of HADRs, and 5–6% of the drugs were associated with anemia, leucopenia, or thrombocytopenia.

Details for any or very common frequencies of HADR and their predictors are presented in Table 1.

**Table 1:** Characteristics of drugs for which marketing authorization was granted by the EMA.

Authorized drugs for human use	Number of MA	Route of administration* N (%)				Additional monitoring N (%)	Generic N (%)	Biosimilar N (%)	Conditional approval N (%)	Exceptional circumstances N (%)	Accelerated assessment N (%)	Orphan medicine N (%)	EML 2023 N (%)
		Enteral	Parenteral	Local	Enteral and parenteral								
Frequency and type of HADR	1349 (100.00)	641 (47.52)	642 (47.59)	43 (3.19)	23 (1.70)	338 (25.06)	232 (17.20)	76 (5.63)	42 (3.11)	37 (2.74)	36 (2.67)	146 (10.82)	387 (28.69)
any frequency of any HADR	739 (54.78)	368 (57.41)	349 (54.36)	5 (11.63)	17 (73.91)	161 (47.63)	166 (71.55)	66 (86.84)	22 (52.38)	12 (32.43)	16 (44.44)	68 (46.58)	278 (71.83)
very common frequency of any HADR	278 (20.61)	98 (15.29)	178 (27.73)	0 (0.00)	2 (8.70)	93 (27.51)	43 (18.53)	37 (48.68)	20 (47.62)	6 (16.22)	11 (30.56)	45 (30.82)	97 (25.06)
any frequency of ANEMIA	546 (40.47)	272 (42.43)	261 (40.65)	2 (4.65)	11 (47.83)	125 (36.98)	124 (53.45)	67 (88.16)	19 (45.24)	8 (21.62)	13 (36.11)	50 (34.25)	231 (59.69)
very common frequency of ANEMIA	199 (14.75)	77 (12.01)	120 (18.69)	0 (0.00)	2 (8.70)	71 (21.01)	32 (13.79)	23 (30.26)	18 (42.86)	5 (13.51)	9 (25.00)	36 (24.66)	69 (17.83)
any frequency of LEUCOPENIA	488 (36.17)	250 (39.00)	227 (35.36)	2 (4.65)	9 (39.13)	106 (31.36)	124 (53.45)	38 (50.00)	17 (40.48)	7 (18.92)	12 (33.33)	51 (34.93)	200 (51.68)
very common frequency of LEUCOPENIA	214 (15.86)	73 (11.39)	139 (21.65)	0 (0.00)	2 (8.70)	72 (21.30)	40 (17.24)	29 (38.16)	15 (35.71)	3 (8.11)	7 (19.44)	34 (23.29)	81 (20.93)
any frequency of THROMBOCYTOPENIA	555 (41.14)	283 (44.15)	261 (40.65)	1 (2.33)	10 (43.48)	110 (32.54)	140 (60.34)	54 (71.05)	16 (38.10)	6 (16.22)	14 (38.89)	50 (34.25)	237 (61.24)
very common frequency of THROMBOCYTOPENIA	173 (12.82)	62 (9.67)	109 (16.98)	0 (0.00)	2 (8.70)	59 (17.46)	31 (13.36)	26 (34.21)	14 (33.33)	4 (10.81)	10 (27.78)	32 (21.92)	67 (17.31)

EML, World Health Organization Essential Medicines List; HADR, Hematological Adverse Drug Reaction; MA, Marketing Authorization.

Two-thirds of all MAs were found in the four ATC groups from the 1st level: A—alimentary tract and metabolism (12.38%), J—antiinfectives for systemic use (14.31%), L—antineoplastic and immunomodulating agents (29.13%), and N—nervous system (11.12%). Drugs from the L group had the highest frequency of drugs requiring additional monitoring (39.64%), generics (29.74%), biosimilars (69.74%), conditionally approved drugs (59.52%), accelerated assessment drugs (36.11%), orphan drugs (34.93%), and EML drugs (33.33%). The A group had the highest number of MAs issued under exceptional circumstances (35.14%).

All members of the P (antiparasitic products, insecticides, and repellents) group of drugs had any HADR or anemia. The L group had the highest frequency of the following HADRs: any frequency of leucopenia (74.81%); any frequency of thrombocytopenia (74.05%); very common frequency of any HADR (60.81%), anemia (46.06%), leucopenia (51.65%), and thrombocytopenia (40.71%). More detailed data about the HADRs according to ATC group can be found in the *Supplementary File.*

Multivariate binary logistic regression revealed that the following regulatory factors increased the odds of any frequency of HADR, anemia, or thrombocytopenia: generic status (odds ratio [OR] 1.76–2.37), biosimilar status (OR 3.26–11.25) and WHO EML status (OR 2.08–2.53). In the case of any frequency of leucopenia, generic status and WHO EML increased the risk by at least two times (OR 2.10 and 2.34, respectively). The only protective factor that decreased the risk of any frequency of anemia by approximately 20% was the route of administration (from highest risk to lowest risk: enteral, parenteral and local routes of administration).

A very common frequency of any HADR, anemia, leucopenia, or thrombocytopenia was increasingly reported among biosimilars, drugs with conditional approval, or drugs listed at the WHO EML. Only very common thrombocytopenia had 3 additional regulatory predictors of increased risk: generic status (OR 1.67), accelerated assessment (OR 3.03), and orphan drug status (OR 1.93). No regulatory predictor was shown to decrease the risk in the case of the very common frequency of HADRs.

**Figure 2:**
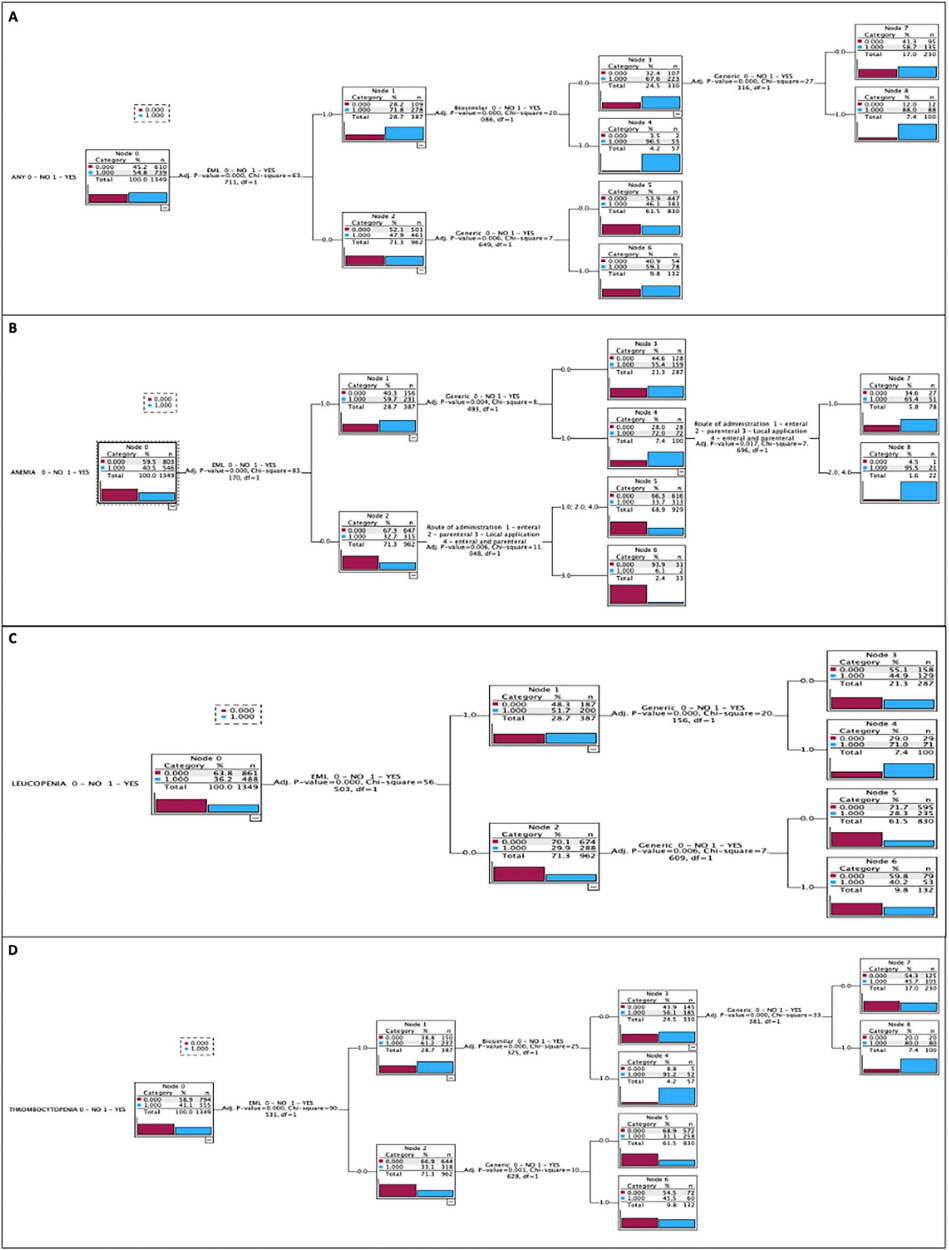
Decision tree showing the effects of a combination of predictors on the probability of hematological adverse drug reactions (HADRs) of any frequency. (**A**) Any HADR; (**B**) ANEMIA; (**C**) LEUCOPENIA; (**D**) THROMBOCYTOPENIA. EML, World Health Organization Essential Medicines List.

**Figure 3:**
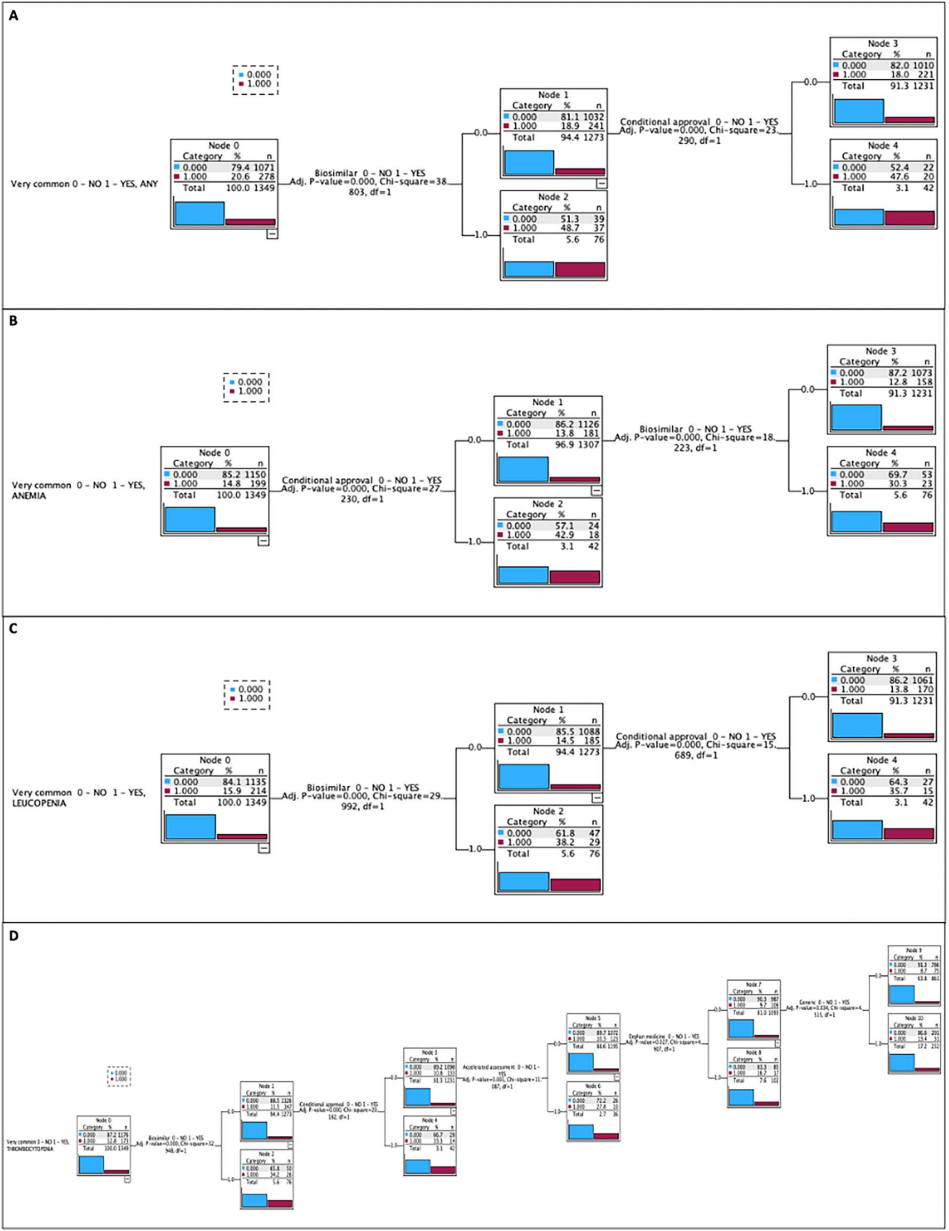
Decision tree showing the effects of a combination of predictors on the probability of hematological adverse drug reactions (HADRs) of very common frequency. (**A**) Any HADR; (**B**) ANEMIA; (**C**) LEUCOPENIA; (**D**) THROMBOCYTOPENIA.

**Figure 4:**
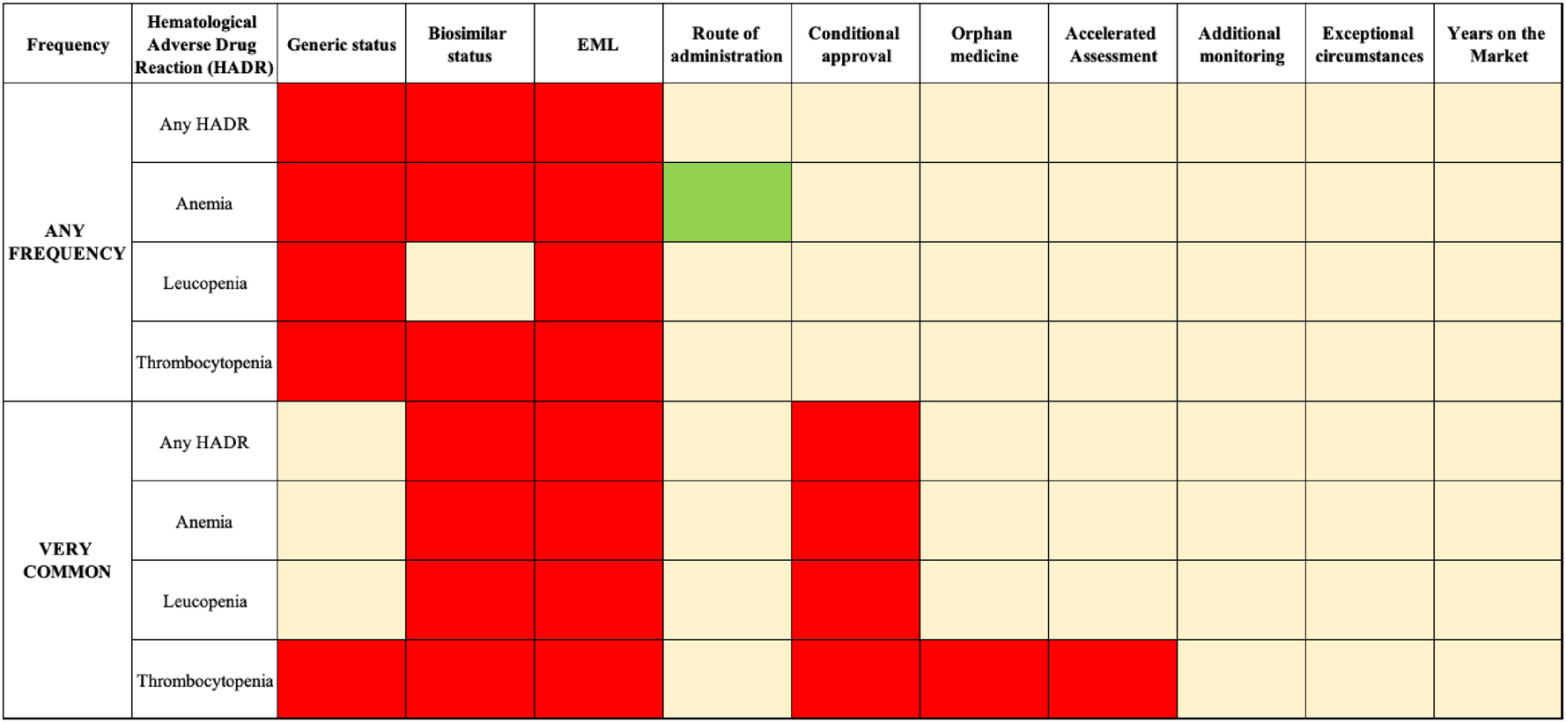
Predictors that were analyzed (red—factors significantly increasing the risk; green—factors significantly decreasing the risk; orange—factors that showed no significance). EML, World Health Organization Essential Medicine List.

Table 2 shows the regulatory factors that increased or decreased the risk of any frequency and very common frequency of any HADR or specific blood disorders analyzed in this study, as well as the features of the logistic regression model. The decision tree of having HADR with any (Fig. 2) or very common frequency (Fig. 3) was satisfactorily differentiated based on the revealed predictors. Figure 4 presents all the predictors that were analyzed.

**Table 2:** Predictors of any and very common frequency of HADR (only significant predictors are shown for the sake of clarity).

Frequency	Hematological Adverse Drug Reaction (HADR)	Factors that increase the risk factor (OR; 95% CI; p value)	Factors that decrease the risk factor (OR; 95% CI; *p* value)	Model features Hosmer and Lemeshow Test (chi-square; df; p value) Cox & Snell R^2^ Nagelkerke’s R^2^	Figures
ANY FREQUENCY	Any HADR	Generic (2.35; [1.65–3.34]; < 0.001) Biosimilar (5.91; [2.90–12.01]; < 0.001) EML (2.08; [1.59–2.74]; < 0.001)	–	11.56; 8; 0.172 0.09 0.12	Fig. 2A
	Anemia	Generic (1.76; [1.29–2.40]; < 0.001) Biosimilar (11.25; [5.41–23.38]; < 0.001) EML (2.32; [1.78–3.01]; < 0.001)	Route of administration (0.79; [0.65–0.96]; 0.016)	12.58; 8; 0.127 0.11 0.15	Fig. 2B
	Leucopenia	Generic (2.10; [1.50–2.94]; < 0.001) EML (2.24; [1.72–2.92]; < 0.001)	–	11.84; 8; 0.158 0.07 0.09	Fig. 2C
	Thrombocytopenia	Generic (2.37; [1.74–3.23]; < 0.001) Biosimilar (3.26; [1.89–5.64]; < 0.001) EML (2.53; [1.94–3.28]; < 0.001)	–	8.41; 5; 0.135 0.10 0.14	Fig. 2D
VERY COMMON	Any HADR	Biosimilar (3.32; [1.94–5.66]; < 0.001) Conditional approval (2.84; [1.41–5.72]; 0.004) EML (1.39; [1.01–1.92]; 0.042)	–	12.39; 8; 0.135 0.05 0.08	Fig. 3A
	Anemia	Biosimilar (2.27; [1.26–4.11]; 0.007) Conditional approval (3.41; [1.65–7.04]; < 0.001) EML (1.47; [1.02–2.11]; 0.038)	–	10.35; 8; 0.241 0.04 0.07	Fig. 3B
	Leucopenia	Biosimilar (2.73; [1.56–4.79]; < 0.001) Conditional approval (2.24; [1.07–4.72]; 0.033) EML (1.59; [1.13–2.26]; 0.009)	–	5.50; 8; 0.704 0.04 0.08	Fig. 3C
	Thrombocytopenia	Generic (1.67; [1.01–2.76]; 0.047) Biosimilar (3.85; [2.12–7.01]; < 0.001) Conditional approval (2.96; [1.36–6.44]; 0.006) Accelerated assessment (3.03; [1.35–6.81]; 0.007) Orphan medicines (1.93; [1.12–3.35]; 0.019) EML (1.57; [1.07–2.30]; 0.020)	–	14.01; 8; 0.081 0.05 0.09	Fig. 3D

EML, World Health Organization Essential Medicine List; Fig, Figure.

### Analysis of INNs

Characteristics of any frequency and very common frequency of HADRs, anemia, leucopenia, and thrombocytopenia at the INN level are shown in Table 3. One-fifth of INNs were listed on the WHO EML, and one-third of the INNs were under additional monitoring. Only 3.62% of the INNs were approved through the accelerated assessment, while approximately 4% of INNs were under conditional approval or exceptional circumstances. Half of the INNs had a HADR of any frequency, and one-fifth of the INNs were associated with HADRs with a very common frequency. One-third of the drugs were associated with any frequency of anemia, leucopenia, or thrombocytopenia, while 13–16% of INNs were associated with a very common frequency of these blood disorders.

**Table 3:** Characteristics of INNs authorized by the EMA.

Authorized drugs for human use	Total number of INN N (%)	Additional monitoring N (%)	Conditional approval N (%)	Exceptional circumstances N (%)	Accelerated assessment N (%)	Orphan medicine N (%)	EML 2023 N (%)
Frequency and type of HADR	883 (100.00)	296 (33.52)	41 (4.64)	36 (4.08)	32 (3.62)	142 (16.08)	197 (22.31)
Any frequency of any HADR	462 (52.32)	130 (43.92)	22 (53.66)	12 (33.33)	14 (43.75)	66 (46.48)	117 (59.39)
Very common frequency of any HADR	191 (21.63)	80 (27.03)	20 (48.78)	6 (16.67)	10 (31.25)	45 (31.69)	39 (19.80)
Any frequency of ANEMIA	327 (37.03)	95 (32.09)	19 (46.34)	8 (22.22)	11 (34.38)	48 (33.80)	89 (45.18)
Very common frequency of ANEMIA	140 (15.86)	65 (21.96)	18 (43.90)	5 (13.89)	8 (25.00)	36 (25.35)	25 (12.69)
Any frequency of LEUCOPENIA	298 (33.75)	90 (30.41)	17 (41.46)	7 (19.44)	10 (31.25)	49 (34.51)	80 (40.61)
Very common frequency of LEUCOPENIA	137 (15.52)	59 (19.93)	15 (36.59)	3 (8.33)	6 (18.75)	34 (23.94)	29 (14.72)
Any frequency of THROMBOCYTOPENIA	320 (36.24)	86 (29.05)	16 (39.02)	6 (16.67)	12 (37.50)	48 (33.80)	87 (44.16)
Very common frequency of THROMBOCYTOPENIA	114 (12.91)	65 (21.96)	14 (34.15)	4 (11.11)	9 (28.13)	32 (22.54)	22 (11.17)

EML, World Health Organization Essential Medicine List; HADR, Hematological Adverse Drug Reaction; INN, International Nonproprietary Name.

The logistic regression for INNs did not reveal any factors that act protectively on the risk of any HADR of any or a very common frequency. WHO EML status was the only regulatory factor that increased the risk by approximately 1.5 times for any frequency of HADR, anemia, leucopenia, or thrombocytopenia. The factors increasing the risk of a very common frequency of any HADR and anemia were mandatory additional monitoring (ORs of 1.48 and 1.67, respectively) and conditional approval (ORs of 2.60 and 3.01, respectively). Conditional approval and orphan status increased the risk of very common leucopenia by 2.13 and 1.71 times, respectively. The risk of very common thrombocytopenia increased when a drug had conditional approval, was approved through accelerated assessment, or was an orphan drug (OR 2.70, 2.75 and 1.77, respectively).

### Analysis of biosimilars and related biologicals

We found 19 unique INNs within biosimilars, with 96 MAs, including their biologicals. All biosimilars were administered parenterally, where two out of three (63.16%) were under additional monitoring, and the majority were on the WHO Essential Medicine List (57.89%). No biosimilar was authorized conditionally or under exceptional circumstances. Only one item was approved via accelerated assessment, and only one item had the status of an orphan drug.

No discrepancy was found in the HADR frequency among biosimilars of the same INN. Only somatropin had a difference in the frequency of HADR, whereas biosimilars had no HADR in comparison to biologicals, which have anemia with an uncommon frequency. Trastuzumab was the only INN associated with a very common frequency of anemia, leucopenia, and thrombocytopenia among the biosimilars. Four out of five authorized biologics (biosimilars + biologicals) had any frequency of HADR or anemia, while three out of four had HADR or anemia at the INN level. Leucopenia was most common (34.38% for the MA level, 21.05% for the INN level). Table 4 shows an overview of the frequencies of any or a specific HADR at the MA or INN level. The biosimilars were found only in six ATC groups (A, B, G, H, L, and S). In the *Supplementary File,* the results of any or specific HADR for biosimilars and their biologicals for all frequencies per ATC group are presented.

**Table 4:** HADR frequency of biosimilars and their biologicals authorized by the EMA.

BIOSIMILARS		Very common N (%)	Common N (%)	Uncommon N (%)	Rare N (%)	Very rare N (%)	Not known N (%)	Any frequency N (%)
INN level	ANY HADR	6 (31.58)	10 (52.63)	7 (36.84)	8 (42.11)	2 (10.53)	3 (15.79)	14 (73.68)
	ANEMIA	3 (15.79)	7 (36.84)	3 (15.79)	7 (36.84)	1 (5.26)	0 (0.00)	14 (73.68)
	LEUCOPENIA	4 (21.05)	4 (21.05)	3 (15.79)	3 (15.79)	0 (0.00)	1 (5.26)	7 (36.84)
	THROMBOCYTOPENIA	4 (21.05)	4 (21.05)	4 (21.05)	4 (21.05)	0 (0.00)	1 (5.26)	10 (52.63)
MA level	ANY HADR	41 (42.71)	61 (63.54)	46 (47.92)	36 (37.50)	10 (10.42)	17 (17.71)	78 (81.25)
	ANEMIA	25 (26.04)	35 (36.46)	20 (20.83)	35 (36.46)	4 (4.17)	0 (0.00)	79 (82.29)
	LEUCOPENIA	33 (34.38)	23 (23.96)	12 (12.50)	20 (20.83)	0 (0.00)	6 (6.25)	45 (46.88)
	THROMBOCYTOPENIA	29 (30.21)	27 (28.13)	23 (23.96)	21 (21.88)	0 (0.00)	7 (7.29)	62 (64.58)

HADR, Hematological Adverse Drug Reaction; INN, International Nonproprietary Name; MA, Marketing Authorization.

In the *Supplementary File*, the checklist is provided, listing all INNs with their respective brand names according to the frequency of anemia, leucopenia, and thrombocytopenia, taking into account only very common occurrences.

## Discussion

Our results showed that any HADR of any frequency was detected in approximately 55% of all MAs; this frequency was greater for drugs with a generic status or biosimilar status or for those listed in the WHO EML. The risk of very common HADRs was significantly increased among drugs with biosimilar status, drugs included on the WHO EML, and drugs with conditional MA.

Our analysis at the level of the INN showed that no factor exerted significant protective effects against all or specific HADRs of any frequency, while WHO EML was a factor that increased the risk of any frequency of all analyzed HADRs.

The majority of the factors associated with HADRs increase the risk (e.g., generic status, biosimilar, WHO EML, conditional approval, additional monitoring, etc.), while only the administration route was a protective factor.

In patients taking biosimilars and their biological drugs, HADRs are very common. Bearing in mind that until recently, i.e., until the position of the EMA in September 2022^19^, biosimilars and biological drugs were not considered mutually interchangeable, as is the case with generic drugs and their reference; therefore, the transition in therapy from one to another biological or biosimilar was not common. Our results showed that there was no difference in the frequency of hematological reactions between biosimilars and their reference biological drug, which is in line with other studies^11^, except in the case of somatropin, where the biosimilar does not have anemia with an unknown frequency listed as HADR while the reference drug does.

For a drug, generic or biosimilar, to be marketed, the data exclusivity of the reference drug must expire so that the drug first receives marketing authorization from the regulatory body and then that the market protection of the reference drug expires for the generic or biosimilar drug to be placed on the market^20,21^. This takes tens of years, depending on the regulations, and during that time, the reference drug is also undergoing postmarketing monitoring. These factors are expected to impact the frequency of HADRs because more real-world data on the type of ADR and its frequency, where generics and biosimilars are marketed, are available, and active substances are being monitored for a more extended period.

The influence of the inclusion of a drug on the WHO EML as a factor can be explained by the requirements that must be met for the drug to be considered for the EML, such as evidence on comparative effectiveness, safety, costs, cost-effectiveness, regulatory status, market availability, and others^22^. To fulfill all the previously mentioned requirements, the drug must be on the market and an active substance used for many years, leading to the well-known safety profile of this drug.

Conditional MA status implies continuous monitoring of the use of the drug (most often, the continuation of clinical trials or mandatory PASSs) and, therefore, highly monitored reporting of ADRs. Studies have shown no increased risk of safety issues for conditionally approved drugs^23^.

Additional monitoring is a factor that increases the risk, which is required when more data on drug safety or efficacy are needed to evaluate the benefit-risk profile worldwide considering long-term use, whether the drug is a new active substance or biological drug, whether it has conditional approval, whether it is approved under exceptional circumstances, or when the MA holder must conduct additional studies or be approved with special obligations^24^. For these drugs to be closely monitored, more pharmacovigilance activities and more real-world data are expected, explaining our study's results^23^.

The orphan status of a drug is also shown to increase the risk of HADR. Considering the rarity and severity of diseases in which patients are treated with these drugs (life-threatening or chronically debilitating), close monitoring of patients receiving orphan drugs is expected, especially given the large amount of data related to ADRs, which may influence the change in the safety profile of drugs for SmPC^25^. One study showed that 70% of drugs authorized by the FDA had changes in labeling due to safety^26^.

Accelerated assessment is used by the EMA when drug approval in shorter review timelines is considered to be of major public health interest or represents a therapeutic innovation^27^. The justification that a drug fulfills one of these conditions is left to the applicant without a clear definition of a major public health interest^28^. Our study showed that accelerated assessment increases the risk of HADR, which is in line with other studies showing the potential relationship between ‘fast track’ or ’priority review’ and postapproval safety issues^23^.

The influence of the administration route on HADRs was also shown in a study conducted by Ingrasciotta et al., where red cell aplasia was linked to the route of administration in the case of epoetin alfa^11^.

### Study limitations

The main limitation of this study was that only drugs authorized through centralized procedures were analyzed. However, this factor was not expected to have a significant impact since the majority of novel drugs are authorized by the EMA. Additional studies analyzing drugs authorized through national procedures (NPs), decentralized procedures (DPs), or mutual recognition procedures (MPRs) are necessary. More drugs are approved through NPs, DPs, and MRPs than through the CP, but drugs authorized by the EMA are available for fewer years on the market (our study showed that half of them have been available for less than 7 years since authorization, which does not mean that they were on the market from the first day), leading to insufficient awareness of the HADR of these drugs. Additionally, we did not consider possible differences in the SmPC between the EMA and other medical agencies, e.g., the Food and Drug Administration (FDA), even though it is known that there may be discrepancies in product information and differences in the decision-making regulatory framework between these two regulators^29–31^ and different safety conclusions between the EU and Canada and the United Kingdom (UK)^32^.

Additionally, we did not perform a statistical analysis in which the predictor was the therapeutic group or drug indication.

### Strengths of the study

To the best of our knowledge, this is the first study investigating the association between regulatory factors and HADRs and the first study using a methodology that completely relies on publicly available data. Ferner et al. explored data available in the SmPC related to monitoring for HADR using the UK’s electronic database^33^. The main strength of our study was that we included all drugs authorized through the CP without therapeutic limitations or authorization date limitations, which was not the case in other studies using the EMA database as the main source of data. Mol et al. excluded biosimilars when conducting a study related to postapproval safety issues with innovative drugs^23^; Francisca RDC et al. analyzed drugs authorized between 2006 and 2017, excluding generics, when analyzing the introduction or discontinuation of additional risk minimization measures (RMMs)^32^; Zeitoun et al. explored the association between regulatory review time and postmarket safety events excluding reformulations, combination therapies, nontherapeutic agents, generics and biosimilars with limitations on authorization dates (2001–2010)^34^; and Meregaglia et al. excluded drugs receiving MA before 2017 when assessing patient-reported outcomes in the authorization by the EMA^31^. We analyzed SmPC data from each of the 1349 authorized MAs for drugs for human use since SmPC is considered a routine RMM, and one study showed that 36% of additional RMMs targeted “blood and blood-forming organs”^31^.

We also added and analyzed the impact of the administration route as well as the presence of a drug on the WHO EML. The additional strength of the study is that it analyzed the HADRs for each MA, INN, and biosimilar and their biological agents.

## Conclusion

There are certain characteristics of drugs that are associated with hematological adverse reactions; they reflect the idiosyncratic nature of the majority of hematological adverse reactions, which require time and widespread use to be recognized a sufficient number of times and then entered the official summary of product characteristics. Knowledge of these characteristics could be of use to clinicians when choosing a drug that should be prescribed to a patient prone to anemia, leucopenia or thrombocytopenia, as well as when an observed hematological adverse reaction should be ascribed to one of numerous drugs the patient was taking simultaneously.

### Supplementary Information

Supplementary Information.

## Data Availability

The data used in the study and other materials not contained in the Supplementary File are available to interested readers from the corresponding author upon reasonable request.

## References

[1] Guideline on good pharmacovigilance practices (GVP) Annex I—Definitions (Rev 4)

[2] Naisbitt DJ, Gordon SF, Pirmohamed M, Park BK (2000). Immunological principles of adverse drug reactions: The initiation and propagation of immune responses elicited by drug treatment. Drug Saf..

[3] Meulenhoff JS (1984). Adverse-effects of drugs on the blood. Pharmaceutisch Weekblad-Sci. Ed..

[4] Knight K, Wade S, Balducci L (2004). Prevalence and outcomes of anemia in cancer: A systematic review of the literature. Am J Med..

[5] Aapro M, Österborg A, Gascón P, Ludwig H, Beguin Y (2012). Prevalence and management of cancer-related anaemia, iron deficiency and the specific role of i.v. iron. Ann. Oncol..

[6] Saint A (2020). Iron deficiency during first-line chemotherapy in metastatic cancers: A prospective epidemiological study. Support Care Cancer.

[7] Wassie M, Aemro A, Fentie B (2021). Prevalence and associated factors of baseline anemia among cervical cancer patients in Tikur Anbesa Specialized Hospital, Ethiopia. BMC Women’s Health.

[8] Olivier P (2002). Assessing the feasibility of using an adverse drug reaction preventability scale in clinical practice: A study in a French emergency department. Drug Saf..

[9] MEMO/08/782, Brussels, 10 December 2008. Strengthening pharmacovigilance to reduce adverse effects of medicine. *European Commission* http://europa.eu/rapid/press-release_MEMO-08-782_en.htm?locale=en. Accessed 8 July 2023.

[10] Giardina C (2018). Adverse drug reactions in hospitalized patients: Results of the FORWARD (Facilitation of Reporting in Hospital Ward) study. Front. Pharmacol..

[11] Ingrasciotta Y (2018). Safety of biologics, including biosimilars: Perspectives on current status and future direction. Drug Saf..

[12] Arnardottir AH (2013). Post-approval safety issues with innovative drugs: A European cohort study. Drug Saf..

[13] Santoro A, Genov G, Spooner A, Raine J, Arlett P (2017). Promoting and protecting public health: How the European Union pharmacovigilance system works. Drug Saf..

[14] Nagai S (2019). Flexible and expedited regulatory review processes for innovative medicines and regenerative medical products in the US, the EU, and Japan. Int. J. Mol. Sci..

[15] Download medicine data. European public assessment reports (EPAR). *EMA* https://www.ema.europa.eu/en/medicines/download-medicine-data. Accessed 30 June 2023.

[16] Public data from Article 57 database. Route of administration. *EMA* https://www.ema.europa.eu/en/human-regulatory/post-authorisation/data-medicines-iso-idmp-standards/public-data-article-57-database. Accessed 05 July 2023.

[17] Web Annex A. World Health Organization Model List of Essential Medicines—23rd List, 2023. In *The Selection and Use of Essential Medicines 2023: Executive Summary of the Report of the 24th WHO Expert Committee on the Selection and Use of Essential Medicines*, 24–28 April 2023 (World Health Organization, 2023) (WHO/MHP/HPS/EML/2023.02). Licence: CC BY-NC-SA 3.0 IGO.

[18] IBM Corp. (2023). IBM SPSS Statistics for Mac (Version 29.0) [Computer software]. IBM Corp.

[19] Statement on the scientific rationale supporting interchangeability of biosimilar medicines in the EU. *EMA* https://www.ema.europa.eu/en/documents/public-statement/statement-scientific-rationale-supporting-interchangeability-biosimilar-medicines-eu_en.pdf. Accessed 20 Sept 2023. 10.1136/ejhpharm-2022-003571 PMC9614114 36283720

[20] Data exclusivity. *EMA* https://www.ema.europa.eu/en/glossary/data-exclusivity. Accessed 20 Sept 2023.

[21] EMA. Market protection. https://www.ema.europa.eu/en/glossary/market-protection. Accessed 20 Sept 2023.

[22] Information for applicants preparing a submission for the 2023 meeting of the WHO Expert Committee on Selection and Use of Essential Medicines. Geneva: World Health Organization; 2022 (WHO/MHP/HPS/EML/2022.01). Licence: CC BY-NC-SA 3.0 IGO.

[23] Mol PG (2013). Post-approval safety issues with innovative drugs: A European cohort study. Drug Saf..

[24] Medicines under additional monitoring. *EMA* https://www.ema.europa.eu/en/human-regulatory/post-authorisation/pharmacovigilance/medicines-under-additional-monitoring. Accessed 15 Sept 2023

[25] Orphan designation. *EMA* https://www.ema.europa.eu/en/human-regulatory/overview/orphan-designation-overview. Accessed 15 Sept 2023.

[26] Fan M (2022). Postmarketing safety of orphan drugs: A longitudinal analysis of the US Food and Drug Administration database between 1999 and 2018. Orphanet J. Rare Dis..

[27] Accelerated assessment. *EMA* https://www.ema.europa.eu/en/human-regulatory/marketing-authorisation/accelerated-assessment. Accessed 15 Sept 2023.

[28] Pre-authorisation guidance. 2.8 Is my product eligible for an accelerated assessment? Rev. July 2023. *EMA* https://www.ema.europa.eu/en/human-regulatory/marketing-authorisation/pre-authorisation-guidance. Accessed 15 Sept 2023.

[29] Wu Y, Xiao W, Tong W, Borlak J, Chen M (2022). A systematic comparison of hepatobiliary adverse drug reactions in FDA and EMA drug labeling reveals discrepancies. Drug Discov. Today.

[30] Dal Pan GJ, Arlett PR (2015). The US Food and Drug Administration-European Medicines Agency collaboration in pharmacovigilance: Common objectives and common challenges. Drug Saf..

[31] Meregaglia M, Malandrini F, Angelini S, Ciani O (2023). The assessment of patient-reported outcomes for the authorisation of medicines in Europe: A review of European Public Assessment Reports from 2017 to 2022. Appl. Health Econ. Health Policy.

[32] Francisca RD (2021). Introduction or discontinuation of additional risk minimisation measures during the life cycle of medicines in Europe. Drug Saf..

[33] Ferner RE, Coleman J, Pirmohamed M, Constable SA, Rouse A (2005). The quality of information on monitoring for haematological adverse drug reactions. Br. J. Clin. Pharmacol..

[34] Zeitoun JD, Lefèvre JH, Downing NS, Bergeron H, Ross JS (2015). Regulatory review time and post-market safety events for novel medicines approved by the EMA between 2001 and 2010: A cross-sectional study. Br. J. Clin. Pharmacol..

