# Supplementary material for: Factors associated with hematological adverse reactions of drugs authorized via the centralized procedure
Source: Sci Rep. 2024 Apr 20;14:9074. doi: 10.1038/s41598-024-59710-3 (PMC11032331; doi:10.1038/s41598-024-59710-3)
Supplement: Supplementary file 1 — Supplementary Information. [file 41598_2024_59710_MOESM1_ESM.docx]

1. **RESULTS SHOWING ALL FREQUENCIES OF ANY OR SPECIFIC HADR PER MARKETING AUTHORIZATION (MA) AND NON-PROPRIETARY NAME (INN)**

| **Frequency** | **Verry common N (%)** | | **Common N (%)** | | **Uncommon N (%)** | | **Rare N (%)** | | **Very rare N (%)** | | **Not known N (%)** | | **Any frequency N (%)** | |
| --- | --- | --- | --- | --- | --- | --- | --- | --- | --- | --- | --- | --- | --- | --- |
| **level** | **MA** | **INN** | **MA** | **INN** | **MA** | **INN** | **MA** | **INN** | **MA** | **INN** | **MA** | **INN** | **MA** | **INN** |
| **Any HADR** | 278 (20.61) | 191 (21.63) | 413 (30.62) | 244 (27.63) | 376 (27.87) | 201 (22.76) | 224 (16.60) | 97 (10.99) | 59 (4.37) | 28 (3.17) | 146 (10.82) | 89 (10.08) | 739 (54.78) | 140 (15.86) |
| **Anemia** | 199 (14.75) | 140 (15.86) | 199 (14.75) | 113 (12.80) | 187 (13.86) | 105 (11.89) | 91 (6.75) | 31 (3.51) | 45 (3.34) | 22 (2.49) | 47 (3.48) | 27 (3.06) | 546 (40.47) | 327 (37.03) |
| **Leucopenia** | 214 (15.86) | 137 (15.52) | 260 (19.27) | 155 (17.55) | 193 (14.31) | 107 (12.12) | 94 (6.97) | 36 (4.08) | 38 (2.82) | 13 (1.47) | 74 (5.49) | 38 (4.30) | 488 (36.17) | 298 (33.75) |
| **Thrombocytopenia** | 173 (12.82) | 114 (12.91) | 169 (12.53) | 93 (10.53) | 201 (14.90) | 97 (10.99) | 128 (9.49) | 50 (5.66) | 35 (2.59) | 12 (1.36) | 70 (5.19) | 44 (4.98) | 555 (41.14) | 320 (36.24) |

HADR – Hematological Adverse Drug Reaction; INN - Non-proprietary name; MA – Marketing Authorization

1. **DETAILED FREQUENCY OF HADR ACCORDING TO ATC GROUP**

**A) Results of all frequencies for any HADR on the Marketing Authorization level per ATC group**

| **ANY HADR** | **Very common N (%)** | **Common N (%)** | **Uncommon N (%)** | **Rare N (%)** | **Very rare N (%)** | **Not known N (%)** | **Any frequency N (%)** |
| --- | --- | --- | --- | --- | --- | --- | --- |
| **A** | 2 (1.20) | 13 (7.78) | 13 (7.78) | 19 (11.38) | 4 (2.40) | 5 (2.99) | 46 (27.54) |
| **B** | 14 (14.00) | 24 (24.00) | 36 (36.00) | 29 (29.00) | 18 (18.00) | 12 (12.00) | 70 (70.00) |
| **C** | 5 (7.25) | 11 (15.94) | 20 (28.99) | 15 (21.74) | 1 (1.45) | 15 (21.74) | 39 (56.52) |
| **D** | 0 (0.00) | 2 (15.38) | 1 (7.69) | 1 (7.69) | 0 (0.00) | 1 (7.69) | 5 (38.46) |
| **G** | 0 (0.00) | 3 (6.38) | 4 (8.51) | 0 (0.00) | 0 (0.00) | 1 (2.13) | 7 (14.89) |
| **H** | 0 (0.00) | 8 (22.86) | 5 (14.29) | 0 (0.00) | 0 (0.00) | 1 (2.86) | 12 (34.29) |
| **J** | 5 (2.59) | 49 (25.39) | 61 (31.61) | 27 (13.99) | 12 (6.22) | 26 (13.47) | 99 (51.30) |
| **L** | 239 (60.81) | 256 (65.14) | 171 (43.51) | 92 (23.41) | 19 (4.83) | 55 (13.99) | 229 (58.27) |
| **M** | 0 (0.00) | 8 (24.24) | 13 (39.39) | 10 (30.30) | 0 (0.00) | 0 (0.00) | 16 (48.48) |
| **N** | 3 (2.00) | 24 (16.00) | 40 (26.67) | 31 (20.67) | 2 (1.33) | 18 (12.00) | 73 (48.67) |
| **P** | 1 (50.00) | 1 (50.00) | 1 (50.00) | 0 (0.00) | 0 (0.00) | 0 (0.00) | 2 (100.00) |
| **R** | 1 (1.75) | 0 (0.00) | 4 (7.02) | 0 (0.00) | 3 (5.26) | 1 (1.75) | 5 (8.77) |
| **S** | 0 (0.00) | 4 (12.90) | 3 (9.68) | 0 (0.00) | 0 (0.00) | 1 (3.23) | 7 (22.58) |
| **V** | 7 (12.73) | 9 (16.36) | 4 (7.27) | 0 (0.00) | 0 (0.00) | 10 (18.18) | 18 (32.73) |
| **Not yet assigned** | 1 (25.00) | 1 (25.00) | 0 (0.00) | 0 (0.00) | 0 (0.00) | 0 (0.00) | 1 (25.00) |

HADR - hematological adverse drug reaction

**B) Results of all frequencies for Anemia on the Marketing Authorization level per ATC group**

| **ANEMIA** | **Very common N (%)** | **Common N (%)** | **Uncommon N (%)** | **Rare N (%)** | **Very rare N (%)** | **Not known N (%)** | **Any frequency N (%)** |
| --- | --- | --- | --- | --- | --- | --- | --- |
| **A** | 1 (0.60) | 6 (3.59) | 8 (4.79) | 0 (0.00) | 4 (2.40) | 2 (1.20) | 18 (10.78) |
| **B** | 1 (1.00) | 16 (16.00) | 5 (5.00) | 6 (6.00) | 17 (17.00) | 0 (0.00) | 43 (43.00) |
| **C** | 3 (4.35) | 8 (11.59) | 12 (17.39) | 1 (1.45) | 3 (4.35) | 19 (27.54) | 36 (52.17) |
| **D** | 0 (0.00) | 0 (0.00) | 0 (0.00) | 0 (0.00) | 0 (0.00) | 0 (0.00) | 0 (0.00) |
| **G** | 0 (0.00) | 3 (6.38) | 1 (2.13) | 0 (0.00) | 0 (0.00) | 0 (0.00) | 4 (8.51) |
| **H** | 0 (0.00) | 9 (25.71) | 3 (8.57) | 0 (0.00) | 0 (0.00) | 0 (0.00) | 11 (31.43) |
| **J** | 3 (1.55) | 19 (9.84) | 43 (22.28) | 6 (3.11) | 11 (5.70) | 3 (1.55) | 64 (33.16) |
| **L** | 181 (46.06) | 114 (29.01) | 91 (23.16) | 60 (15.27) | 8 (2.04) | 12 (3.05) | 294 (74.81) |
| **M** | 0 (0.00) | 8 (24.24) | 6 (18.18) | 10 (30.30) | 0 (0.00) | 0 (0.00) | 17 (51.52) |
| **N** | 2 (1.33) | 8 (5.33) | 14 (9.33) | 8 (5.33) | 2 (1.33) | 2 (1.33) | 36 (24.00) |
| **P** | 1 (50.00) | 1 (50.00) | 0 (0.00) | 0 (0.00) | 0 (0.00) | 0 (0.00) | 2 (100.00) |
| **R** | 0 (0.00) | 0 (0.00) | 0 (0.00) | 0 (0.00) | 0 (0.00) | 0 (0.00) | 0 (0.00) |
| **S** | 0 (0.00) | 4 (12.90) | 2 (6.45) | 0 (0.00) | 0 (0.00) | 0 (0.00) | 6 (19.35) |
| **V** | 6 (10.91) | 3 (5.45) | 2 (3.64) | 0 (0.00) | 0 (0.00) | 9 (16.36) | 14 (25.45) |
| **Not yet assigned** | 1 (25.00) | 0 (0.00) | 0 (0.00) | 0 (0.00) | 0 (0.00) | 0 (0.00) | 1 (25.00) |

**C) Results of all frequencies for Leucopenia on the Marketing Authorization level per ATC group**

| **LEUCOPENIA** | **Very common N (%)** | **Common N (%)** | **Uncommon N (%)** | **Rare N (%)** | **Very rare N (%)** | **Not known N (%)** | **Any frequency N (%)** |
| --- | --- | --- | --- | --- | --- | --- | --- |
| **A** | 1 (0.60) | 4 (2.40) | 6 (3.59) | 6 (3.59) | 4 (2.40) | 0 (0.00) | 16 (9.58) |
| **B** | 0 (0.00) | 3 (3.00) | 20 (20.00) | 17 (17.00) | 17 (17.00) | 2 (2.00) | 23 (23.00) |
| **C** | 1 (1.45) | 2 (2.90) | 2 (2.90) | 2 (2.90) | 7 (10.14) | 18 (26.09) | 14 (20.29) |
| **D** | 0 (0.00) | 0 (0.00) | 1 (7.69) | 1 (7.69) | 0 (0.00) | 1 (7.69) | 2 (15.38) |
| **G** | 0 (0.00) | 0 (0.00) | 1 (2.13) | 0 (0.00) | 0 (0.00) | 0 (0.00) | 1 (2.13) |
| **H** | 0 (0.00) | 1 (2.86) | 3 (8.57) | 0 (0.00) | 0 (0.00) | 0 (0.00) | 3 (8.57) |
| **J** | 2 (1.04) | 34 (17.62) | 1 (0.52) | 9 (4.66) | 1 (0.52) | 8 (4.15) | 61 (31.61) |
| **L** | 203 (51.65) | 189 (48.09) | 89 (22.65) | 30 (7.63) | 7 (1.78) | 19 (4.83) | 294 (74.81) |
| **M** | 0 (0.00) | 0 (0.00) | 7 (21.21) | 10 (30.30) | 0 (0.00) | 0 (0.00) | 10 (30.30) |
| **N** | 1 (0.67) | 16 (10.67) | 28 (18.67) | 19 (12.67) | 2 (1.33) | 18 (12.00) | 49 (32.67) |
| **P** | 0 (0.00) | 1 (50.00) | 0 (0.00) | 0 (0.00) | 0 (0.00) | 0 (0.00) | 1 (50.00) |
| **R** | 0 (0.00) | 0 (0.00) | 3 (5.26) | 0 (0.00) | 0 (0.00) | 0 (0.00) | 3 (5.26) |
| **S** | 0 (0.00) | 0 (0.00) | 1 (3.23) | 0 (0.00) | 0 (0.00) | 0 (0.00) | 1 (3.23) |
| **V** | 6 (10.91) | 9 (16.36) | 1 (1.82) | 0 (0.00) | 0 (0.00) | 8 (14.55) | 9 (16.36) |
| **Not yet assigned** | 0 (0.00) | 1 (25.00) | 0 (0.00) | 0 (0.00) | 0 (0.00) | 0 (0.00) | 1 (25.00) |

**D) Results of all frequencies for Thrombocytopenia on the Marketing Authorization level per ATC group**

| **THROMBOCYTOPENIA** | **Very common N (%)** | **Common N (%)** | **Uncommon N (%)** | **Rare N (%)** | **Very rare N (%)** | **Not known N (%)** | **Any frequency N (%)** |
| --- | --- | --- | --- | --- | --- | --- | --- |
| **A** | 0 (0.00) | 8 (4.79) | 1 (0.60) | 14 (8.38) | 4 (2.40) | 2 (1.20) | 28 (16.77) |
| **B** | 0 (0.00) | 9 (9.00) | 26 (26.00) | 4 (4.00) | 19 (19.00) | 4 (4.00) | 39 (39.00) |
| **C** | 2 (2.90) | 2 (2.90) | 2 (2.90) | 15 (21.74) | 4 (5.80) | 12 (17.39) | 36 (52.17) |
| **D** | 0 (0.00) | 0 (0.00) | 1 (7.69) | 0 (0.00) | 0 (0.00) | 1 (7.69) | 2 (15.38) |
| **G** | 0 (0.00) | 0 (0.00) | 4 (8.51) | 0 (0.00) | 0 (0.00) | 0 (0.00) | 4 (8.51) |
| **H** | 0 (0.00) | 1 (2.86) | 2 (5.71) | 0 (0.00) | 0 (0.00) | 1 (2.86) | 3 (8.57) |
| **J** | 2 (1.04) | 16 (8.29) | 30 (15.54) | 12 (6.22) | 2 (1.04) | 19 (9.84) | 74 (38.34) |
| **L** | 160 (40.71) | 128 (32.57) | 105 (26.72) | 47 (11.96) | 1 (0.25) | 15 (3.82) | 291 (74.05) |
| **M** | 0 (0.00) | 1 (3.03) | 8 (24.24) | 10 (30.30) | 0 (0.00) | 0 (0.00) | 12 (36.36) |
| **N** | 1 (0.67) | 1 (0.67) | 21 (14.00) | 26 (17.33) | 2 (1.33) | 8 (5.33) | 49 (32.67) |
| **P** | 0 (0.00) | 1 (50.00) | 0 (0.00) | 0 (0.00) | 0 (0.00) | 0 (0.00) | 1 (50.00) |
| **R** | 1 (1.75) | 0 (0.00) | 0 (0.00) | 0 (0.00) | 3 (5.26) | 1 (1.75) | 5 (8.77) |
| **S** | 0 (0.00) | 0 (0.00) | 0 (0.00) | 0 (0.00) | 0 (0.00) | 0 (0.00) | 0 (0.00) |
| **V** | 7 (12.73) | 3 (5.45) | 1 (1.82) | 0 (0.00) | 0 (0.00) | 7 (12.73) | 11 (20.00) |
| **Not yet assigned** | 0 (0.00) | 0 (0.00) | 0 (0.00) | 0 (0.00) | 0 (0.00) | 0 (0.00) | (0.00) |

1. **RESULTS OF ANY OR SPECIFIC HADR FOR BIOSIMILARS AND THEIR BIOLOGICALS FOR ALL FREQUENCIES PER ATC GROUP**

| **ATC group** | **HADR TYPE** | **Very common N (%)** | **Common N (%)** | **Uncommon N (%)** | **Rare N (%)** | **Very rare N (%)** | **Not known N (%)** | **Any frequency N (%)** |
| --- | --- | --- | --- | --- | --- | --- | --- | --- |
| **A N = 6** | **ANY HADR** | 0 (0.00) | 0 (0.00) | 0 (0.00) | 0 (0.00) | 0 (0.00) | 0 (0.00) | 0 (0.00) |
|  | **ANEMIA** | 0 (0.00) | 0 (0.00) | 0 (0.00) | 0 (0.00) | 0 (0.00) | 0 (0.00) | 0 (0.00) |
|  | **LEUCOPENIA** | 0 (0.00) | 0 (0.00) | 0 (0.00) | 0 (0.00) | 0 (0.00) | 0 (0.00) | 0 (0.00) |
|  | **THROMBOCYTOPENIA** | 0 (0.00) | 0 (0.00) | 0 (0.00) | 0 (0.00) | 0 (0.00) | 0 (0.00) | 0 (0.00) |
| **B N = 6** | **ANY HADR** | 1 (16.67) | 1 (16.67) | 0 (0.00) | 6 (100.00) | 0 (0.00) | 0 (0.00) | 6 (100.00) |
|  | **ANEMIA** | 0 (0.00) | 1 (16.67) | 0 (0.00) | 5 (83.33) | 0 (0.00) | 0 (0.00) | 6 (100.00) |
|  | **LEUCOPENIA** | 0 (0.00) | 0 (0.00) | 0 (0.00) | 0 (0.00) | 0 (0.00) | 0 (0.00) | 0 (0.00) |
|  | **THROMBOCYTOPENIA** | 0 (0.00) | 1 (16.67) | 0 (0.00) | 1 (16.67) | 0 (0.00) | 0 (0.00) | 1 (16.67) |
| **G N = 2** | **ANY HADR** | 0 (0.00) | 0 (0.00) | 0 (0.00) | 0 (0.00) | 0 (0.00) | 0 (0.00) | 0 (0.00) |
|  | **ANEMIA** | 0 (0.00) | 0 (0.00) | 0 (0.00) | 0 (0.00) | 0 (0.00) | 0 (0.00) | 0 (0.00) |
|  | **LEUCOPENIA** | 0 (0.00) | 0 (0.00) | 0 (0.00) | 0 (0.00) | 0 (0.00) | 0 (0.00) | 0 (0.00) |
|  | **THROMBOCYTOPENIA** | 0 (0.00) | 0 (0.00) | 0 (0.00) | 0 (0.00) | 0 (0.00) | 0 (0.00) | 0 (0.00) |
| **H N = 6** | **ANY HADR** | 0 (0.00) | 4 (66.67) | 0 (0.00) | 0 (0.00) | 0 (0.00) | 0 (0.00) | 4 (66.67) |
|  | **ANEMIA** | 0 (0.00) | 5 (83.33) | 0 (0.00) | 0 (0.00) | 0 (0.00) | 0 (0.00) | 5 (83.33) |
|  | **LEUCOPENIA** | 0 (0.00) | 0 (0.00) | 0 (0.00) | 0 (0.00) | 0 (0.00) | 0 (0.00) | 0 (0.00) |
|  | **THROMBOCYTOPENIA** | 0 (0.00) | 0 (0.00) | 0 (0.00) | 0 (0.00) | 0 (0.00) | 0 (0.00) | 0 (0.00) |
| **L N = 53** | **ANY HADR** | 36 (67.92) | 44 (83.02) | 39 (73.58) | 26 (49.06) | 8 (15.09) | 14 (26.42) | 53 (100.00) |
|  | **ANEMIA** | 23 (43.40) | 19 (35.85) | 16 (30.19) | 26 (49.06) | 3 (5.66) | 0 (0.00) | 53 (100.00) |
|  | **LEUCOPENIA** | 29 (54.72) | 19 (35.85) | 9 (16.98) | 17 (32.08) | 0 (0.00) | 5 (9.43) | 38 (71.70) |
|  | **THROMBOCYTOPENIA** | 26 (49.06) | 23 (43.40) | 19 (35.85) | 17 (32.08) | 0 (0.00) | 6 (11.32) | 53 (100.00) |
| **S N = 3** | **ANY HADR** | 0 (0.00) | 3 (100.00) | 0 (0.00) | 0 (0.00) | 0 (0.00) | 0 (0.00) | 3 (100.00) |
|  | **ANEMIA** | 0 (0.00) | 3 (100.00) | 0 (0.00) | 0 (0.00) | 0 (0.00) | 0 (0.00) | 3 (100.00) |
|  | **LEUCOPENIA** | 0 (0.00) | 0 (0.00) | 0 (0.00) | 0 (0.00) | 0 (0.00) | 0 (0.00) | 0 (0.00) |
|  | **THROMBOCYTOPENIA** | 0 (0.00) | 0 (0.00) | 0 (0.00) | 0 (0.00) | 0 (0.00) | 0 (0.00) | 0 (0.00) |

1. **CHECKLIST OF ALL INNs WITH VERY COMMON FREQUENCY OF ANY OR SPECIFIC HADR**

**A) I INNs & TRADE/BRAND NAMES OF DRUGS with ANEMIA with very common frequency**

| **INN beginning letter** | **INN** | **Trade/Brand names** |
| --- | --- | --- |
| **A** | 5-aminolevulinic acid hydrochloride | Gliolan Ameluz |
|  | abemaciclib | Verzenios |
|  | acalabrutinib | Calquence |
|  | adalimumab | Hefiya Hyrimoz Imraldi Hukyndra Yuflyma Amgevita Libmyris Humira Hulio Idacio Amsparity |
|  | alectinib | Alecensa |
|  | alpelisib | Piqray |
|  | ambrisentan | Volibris Ambrisentan Mylan |
|  | artesunate | Artesunate Amivas |
|  | asciminib | Scemblix |
|  | atezolizumab | Tecentriq |
|  | autologous CD34+ enriched cell fraction that contains CD34+ cells transduced with retroviral vector that encodes for the human ADA cDNA sequence | Strimvelis |
|  | avapritinib | Ayvakyt |
|  | avelumab | Bavencio |
|  | axicabtagene ciloleucel | Yescarta |
|  | azacitidine | Vidaza Azacitidine Accord Azacitidine Mylan Azacitidine betapharm Onureg |
| **B** | basiliximab | Simulect |
|  | belantamab mafodotin | Blenrep |
|  | belatacept | Nulojix |
|  | binimetinib | Mektovi |
|  | blinatumomab | Blincyto |
|  | bortezomib | Bortezomib Accord Bortezomib Hospira Bortezomib Sun Bortezomib Fresenius Kabi Velcade |
|  | bosutinib | Bosulif |
|  | brentuximab vedotin | Adcetris |
|  | Brexucabtagene autoleucel | Tecartus |
|  | brigatinib | Alunbrig |
|  | bupivacaine | Zynrelef Exparel liposomal |
|  | busulfan | Busilvex Busulfan Fresenius Kabi |
| **C** | cabazitaxel | Cabazitaxel Accord Jevtana |
|  | cabozantinib | Cabometyx  Cometriq |
|  | capecitabine | Capecitabine Accord Capecitabine Medac Capecitabine Teva Ecansya (previously Capecitabine Krka) Xeloda |
|  | carfilzomib | Kyprolis |
|  | cemiplimab | Libtayo |
|  | ceritinib | Zykadia |
|  | ciltacabtagene autoleucel | Carvykti |
|  | cladribine | Mavenclad Litak |
|  | cobimetinib | Cotellic |
|  | crizotinib | Xalkori |
| **D** | daratumumab | Darzalex |
|  | dasatinib (anhydrous) | Sprycel |
|  | decitabine | Dacogen |
|  | dinutuximab beta | Qarziba (previously Dinutuximab beta EUSA and Dinutuximab beta Apeiron) |
|  | docetaxel | Docetaxel Accord Docetaxel Kabi Taxotere |
|  | dostarlimab | Jemperli |
|  | doxorubicin | Caelyx pegylated liposomal Celdoxome pegylated liposomal Myocet liposomal (previously Myocet) Zolsketil pegylated liposomal |
|  | durvalumab | Imfinzi |
|  | duvelisib | Copiktra |
| **E** | eladocagene exuparvovec | Upstaza |
|  | eltrombopag | Revolade |
|  | encorafenib | Braftovi |
|  | enfortumab vedotin | Padcev |
|  | entrectinib | Rozlytrek |
|  | eribulin | Halaven |
|  | everolimus | Afinitor Votubia |
| **F** | fedratinib | Inrebic |
|  | filgrastim | Accofil Filgrastim Hexal Grastofil Nivestim Ratiograstim Tevagrastim Zarzio |
|  | fulvestrant | Faslodex Fulvestrant Mylan |
| **G** | gemtuzumab ozogamicin | Mylotarg |
|  | glasdegib | Daurismo |
| **I** | ibritumomab tiuxetan | Zevalin |
|  | idecabtagene vicleucel | Abecma |
|  | imatinib | Glivec Imatinib Accord Imatinib Koanaa Imatinib Teva |
|  | inotersen | Tegsedi |
|  | inotuzumab ozogamicin | Besponsa |
|  | interferon beta-1a | Avonex Rebif |
|  | ipilimumab | Yervoy |
|  | irinotecan hydrochloride trihydrate | Onivyde pegylated liposomal (previously known as Onivyde) |
|  | ivosidenib | Tibsovo |
| **L** | larotrectinib | Vitrakvi |
|  | lenalidomide | Lenalidomide Accord Lenalidomide Krka (previously Lenalidomide Krka d.d. Novo mesto) Lenalidomide Krka d.d. Lenalidomide Krka d.d. Novo mesto (previously Lenalidomide Krka) Lenalidomide Mylan Revlimid |
|  | lenvatinib | Kisplyx Lenvima |
|  | lisocabtagene maraleucel | Breyanzi |
|  | loncastuximab tesirine | Zynlonta |
|  | lorlatinib | Lorviqua |
|  | lutetium (177 Lu) chloride | EndolucinBeta Lumark Lutetium (177Lu) chloride Billev (previously Illuzyce) |
|  | lutetium (177Lu) oxodotreotide | Lutathera |
|  | lutetium (177Lu) vipivotide tetraxetan | Pluvicto |
| **M** | macitentan | Opsumit |
|  | melphalan hydrochloride | Phelinun |
|  | melphalan flufenamide | Pepaxti |
|  | mifamurtide | Mepact |
|  | mosunetuzumab | Lunsumio |
|  | mycophenolate mofetil | CellCept Myclausen Mycophenolate mofetil Teva Myfenax |
| **N** | nelarabine | Atriance |
|  | nilotinib | Tasigna |
|  | niraparib | Zejula |
|  | niraparib, abiraterone acetate | Akeega |
|  | nivolumab | Opdivo |
| **O** | obinutuzumab | Gazyvaro |
|  | olaparib | Lynparza |
| **P** | paclitaxel | Abraxane Apealea Pazenir |
|  | palbociclib | Ibrance |
|  | panitumumab | Vectibix |
|  | panobinostat | Farydak |
|  | pembrolizumab | Keytruda |
|  | pertuzumab | Perjeta |
|  | pertuzumab, trastuzumab | Phesgo |
|  | pixantrone dimaleate | Pixuvri |
|  | polatuzumab vedotin | Polivy |
|  | pomalidomide | Imnovid (previously Pomalidomide Celgene) |
|  | ponatinib | Iclusig |
|  | pralsetinib | Gavreto |
|  | pretomanid | Dovprela (previously Pretomanid FGK) |
| **R** | ramucirumab | Cyramza |
|  | regorafenib | Stivarga |
|  | relatlimab / nivolumab | Opdualag |
|  | ribavirin | Rebetol |
|  | ribociclib | Kisqali |
|  | rucaparib | Rubraca |
|  | ruxolitinib | Jakavi Opzelura |
| **S** | sacituzumab govitecan | Trodelvy |
|  | selinexor | Nexpovio |
|  | sirolimus | Hyftor Rapamune |
|  | sofosbuvir | Sovaldi |
|  | sotorasib | Lumykras |
|  | sunitinib | Sunitinib Accord Sutent |
| **T** | tabelecleucel | Ebvallo |
|  | tafasitamab | Minjuvi |
|  | tagraxofusp | Elzonris |
|  | talazoparib | Talzenna |
|  | teclistamab | Tecvayli |
|  | tegafur, gimeracil, oteracil | Teysuno |
|  | temsirolimus | Torisel |
|  | thalidomide | Thalidomide BMS (previously Thalidomide Celgene) Thalidomide Lipomed |
|  | thiotepa | Tepadina Thiotepa Riemser |
|  | tisagenlecleucel | Kymriah |
|  | topotecan | Hycamtin Potactasol Topotecan Hospira |
|  | trabectedin | Yondelis |
|  | trastuzumab | Herceptin Herzuma Kanjinti Ogivri Ontruzant Trazimera Zercepac |
|  | trastuzumab deruxtecan | Enhertu |
|  | trastuzumab emtansine | Kadcyla |
|  | tremelimumab | Imjudo Tremelimumab AstraZeneca |
|  | treosulfan | Trecondi |
|  | trifluridine, tipiracil | Lonsurf |
| **V** | venetoclax | Venclyxto |
|  | vinflunine | Javlor |
|  | voclosporin | Lupkynis |
| **Z** | zanubrutinib | Brukinsa |

**B) INNs & TRADE/BRAND NAMES OF DRUGS with LEUCOPENIA with very common frequency**

| **INN beginning letter** | **INN** | **Trade/Brand names** |
| --- | --- | --- |
| **A** | abemaciclib | Verzenios |
|  | acalabrutinib | Calquence |
|  | adalimumab | Hefiya Hyrimoz Imraldi Hukyndra Yuflyma Amgevita Libmyris Humira Hulio Idacio Amsparity |
|  | aflibercept | Eylea Zaltrap |
|  | alemtuzumab | Lemtrada |
|  | alpelisib | Piqray |
|  | asciminib | Scemblix |
|  | atezolizumab | Tecentriq |
|  | Autologous CD34+ cells encoding ARSA gene | Libmeldy |
|  | autologous CD34+ enriched cell fraction that contains CD34+ cells transduced with retroviral vector that encodes for the human ADA cDNA sequence | Strimvelis |
|  | avapritinib | Ayvakyt |
|  | axicabtagene ciloleucel | Yescarta |
|  | azacitidine | Vidaza Azacitidine Accord Azacitidine Mylan Azacitidine betapharm Onureg |
|  | azathioprine | Jayempi |
| **B** | belantamab mafodotin | Blenrep |
|  | belatacept | Nulojix |
|  | bevacizumab | Abevmy Alymsys Avastin Aybintio Mvasi Onbevzi Oyavas Vegzelma Zirabev |
|  | bexarotene | Targretin |
|  | blinatumomab | Blincyto |
|  | bortezomib | Bortezomib Accord Bortezomib Hospira Bortezomib Sun Bortezomib Fresenius Kabi Velcade |
|  | bosutinib | Bosulif |
|  | brentuximab vedotin | Adcetris |
|  | Brexucabtagene autoleucel | Tecartus |
|  | brigatinib | Alunbrig |
|  | busulfan | Busilvex Busulfan Fresenius Kabi |
| **C** | cabazitaxel | Cabazitaxel Accord Jevtana |
|  | capecitabine | Capecitabine Accord Capecitabine Medac Capecitabine Teva Ecansya (previously Capecitabine Krka) Xeloda |
|  | carfilzomib | Kyprolis |
|  | cemiplimab | Libtayo |
|  | ciltacabtagene autoleucel | Carvykti |
|  | cladribine | Mavenclad Litak |
|  | clofarabine | Evoltra Ivozall |
|  | crizotinib | Xalkori |
| **D** | daratumumab | Darzalex |
|  | dasatinib (anhydrous) | Sprycel |
|  | daunorubicin, cytarabine | Vyxeos liposomal (previously known as Vyxeos) |
|  | decitabine | Dacogen |
|  | dimethyl fumarate | Dimethyl fumarate Accord Dimethyl fumarate Mylan Dimethyl fumarate Neuraxpharm Dimethyl fumarate Polpharma Dimethyl fumarate Teva Skilarence Tecfidera |
|  | dinutuximab beta | Qarziba (previously Dinutuximab beta EUSA and Dinutuximab beta Apeiron) |
|  | docetaxel | Docetaxel Accord Docetaxel Kabi Taxotere |
|  | doxorubicin | Caelyx pegylated liposomal Celdoxome pegylated liposomal Myocet liposomal (previously Myocet) Zolsketil pegylated liposomal |
|  | durvalumab | Imfinzi |
|  | duvelisib | Copiktra |
| **E** | entrectinib | Rozlytrek |
|  | eribulin | Halaven |
| **F** | fedratinib | Inrebic |
|  | fulvestrant | Faslodex Fulvestrant Mylan |
| **G** | gemtuzumab ozogamicin | Mylotarg |
|  | glasdegib | Daurismo |
|  | glibenclamide | Amglidia |
| **H** | hydroxycarbamide | Siklos Xromi |
| **I** | ibritumomab tiuxetan | Zevalin |
|  | ibrutinib | Imbruvica |
|  | ibuprofen | Pedea |
|  | idecabtagene vicleucel | Abecma |
|  | idelalisib | Zydelig |
|  | imatinib | Glivec Imatinib Accord Imatinib Koanaa Imatinib Teva |
|  | inotuzumab ozogamicin | Besponsa |
|  | interferon beta-1a | Avonex Rebif |
|  | interferon beta-1b | Betaferon Extavia |
|  | ipilimumab | Yervoy |
|  | irinotecan hydrochloride trihydrate | Onivyde pegylated liposomal (previously known as Onivyde) |
|  | isatuximab | Sarclisa |
|  | ivosidenib | Tibsovo |
|  | ixazomib | Ninlaro |
| **L** | larotrectinib | Vitrakvi |
|  | lenalidomide | Lenalidomide Accord Lenalidomide Krka (previously Lenalidomide Krka d.d. Novo mesto) Lenalidomide Krka d.d. Lenalidomide Krka d.d. Novo mesto (previously Lenalidomide Krka) Lenalidomide Mylan Revlimid |
|  | lenvatinib | Kisplyx Lenvima |
|  | lisocabtagene maraleucel | Breyanzi |
|  | loncastuximab tesirine | Zynlonta |
|  | lutetium (177 Lu) chloride | EndolucinBeta Lumark Lutetium (177Lu) chloride Billev (previously Illuzyce) |
|  | lutetium (177Lu) oxodotreotide | Lutathera |
|  | lutetium (177Lu) vipivotide tetraxetan | Pluvicto |
| **M** | melphalan flufenamide | Pepaxti |
|  | melphalan hydrochloride | Phelinun |
|  | mercaptopurine | Xaluprine (previously Mercaptopurine Nova Laboratories) |
|  | midostaurin | Rydapt |
|  | mitotane | Lysodren |
|  | mosunetuzumab | Lunsumio |
|  | mycophenolate mofetil | CellCept Myclausen Mycophenolate mofetil Teva Myfenax |
| **N** | nelarabine | Atriance |
|  | nintedanib | Ofev Vargatef |
|  | niraparib | Zejula |
|  | niraparib, abiraterone acetate | Akeega |
|  | nivolumab | Opdivo |
| **O** | obinutuzumab | Gazyvaro |
|  | olaparib | Lynparza |
| **P** | paclitaxel | Abraxane Apealea Pazenir |
|  | palbociclib | Ibrance |
|  | panobinostat | Farydak |
|  | pazopanib | Votrient |
|  | pegaspargase | Oncaspar |
|  | pembrolizumab | Keytruda |
|  | pemetrexed | Alimta Armisarte (previously Pemetrexed Actavis) Ciambra Pemetrexed Accord Pemetrexed Baxter Pemetrexed Fresenius Kabi Pemetrexed Krka Pemetrexed medac Pemetrexed Pfizer (previously Pemetrexed Hospira) Pemetrexed Sandoz |
|  | pertuzumab | Perjeta |
|  | pertuzumab, trastuzumab | Phesgo |
|  | pixantrone dimaleate | Pixuvri |
|  | polatuzumab vedotin | Polivy |
|  | pomalidomide | Imnovid (previously Pomalidomide Celgene) |
|  | pralsetinib | Gavreto |
| **R** | ramucirumab | Cyramza |
|  | relatlimab / nivolumab | Opdualag |
|  | ribavirin | Rebetol |
|  | ribociclib | Kisqali |
|  | rituximab | Blitzima MabThera Rixathon Riximyo Ruxience Truxima |
|  | rucaparib | Rubraca |
|  | ruxolitinib | Jakavi Opzelura |
| **S** | sacituzumab govitecan | Trodelvy |
|  | sarilumab | Kevzara |
|  | selinexor | Nexpovio |
|  | siltuximab | Sylvant |
|  | sirolimus | Hyftor Rapamune |
|  | sofosbuvir | Sovaldi |
|  | sunitinib | Sunitinib Accord Sutent |
| **T** | tafasitamab | Minjuvi |
|  | talazoparib | Talzenna |
|  | teclistamab | Tecvayli |
|  | tegafur, gimeracil, oteracil | Teysuno |
|  | temsirolimus | Torisel |
|  | thalidomide | Thalidomide BMS (previously Thalidomide Celgene) Thalidomide Lipomed |
|  | thiotepa | Tepadina Thiotepa Riemser |
|  | tisagenlecleucel | Kymriah |
|  | topotecan | Hycamtin Potactasol Topotecan Hospira |
|  | trabectedin | Yondelis |
|  | trastuzumab | Herceptin Herzuma Kanjinti Ogivri Ontruzant Trazimera Zercepac |
|  | trastuzumab deruxtecan | Enhertu |
|  | tremelimumab | Imjudo Tremelimumab AstraZeneca |
|  | treosulfan | Trecondi |
|  | trifluridine, tipiracil | Lonsurf |
| **V** | venetoclax | Venclyxto |
|  | vinflunine | Javlor |
| **Z** | zanubrutinib | Brukinsa |

**C) INNs & TRADE/BRAND NAMES OF DRUGS with THROMBOCYTOPENIA with very common frequency**

| **INN beginning letter** | **INN** | **Trade/Brand names** |
| --- | --- | --- |
| **A** | 5-aminolevulinic acid hydrochloride | Gliolan Ameluz |
|  | abemaciclib | Verzenios |
|  | aflibercept | Eylea Zaltrap |
|  | alpelisib | Piqray |
|  | asciminib | Scemblix |
|  | atezolizumab | Tecentriq |
|  | avapritinib | Ayvakyt |
|  | axicabtagene ciloleucel | Yescarta |
|  | azacitidine | Vidaza Azacitidine Accord Azacitidine Mylan Azacitidine betapharm Onureg |
| **B** | belantamab mafodotin | Blenrep |
|  | bevacizumab | Abevmy Alymsys Avastin Aybintio Mvasi Onbevzi Oyavas Vegzelma Zirabev |
|  | blinatumomab | Blincyto |
|  | bortezomib | Bortezomib Accord Bortezomib Hospira Bortezomib Sun Bortezomib Fresenius Kabi Velcade |
|  | bosutinib | Bosulif |
|  | Brexucabtagene autoleucel | Tecartus |
|  | busulfan | Busilvex Busulfan Fresenius Kabi |
| **C** | cabazitaxel | Cabazitaxel Accord Jevtana |
|  | cabozantinib | Cabometyx  Cometriq |
|  | capecitabine | Capecitabine Accord Capecitabine Medac Capecitabine Teva Ecansya (previously Capecitabine Krka) Xeloda |
|  | carfilzomib | Kyprolis |
|  | cemiplimab | Libtayo |
|  | ciltacabtagene autoleucel | Carvykti |
|  | cladribine | Mavenclad Litak |
|  | crizotinib | Xalkori |
| **D** | daratumumab | Darzalex |
|  | dasatinib (anhydrous) | Sprycel |
|  | decitabine | Dacogen |
|  | dinutuximab beta | Qarziba (previously Dinutuximab beta EUSA and Dinutuximab beta Apeiron) |
|  | docetaxel | Docetaxel Accord Docetaxel Kabi Taxotere |
|  | doxorubicin | Caelyx pegylated liposomal Celdoxome pegylated liposomal Myocet liposomal (previously Myocet) Zolsketil pegylated liposomal |
|  | durvalumab | Imfinzi |
|  | duvelisib | Copiktra |
| **F** | fedratinib | Inrebic |
|  | filgrastim | Accofil Filgrastim Hexal Grastofil Nivestim Ratiograstim Tevagrastim Zarzio |
|  | fulvestrant | Faslodex Fulvestrant Mylan |
| **G** | gemtuzumab ozogamicin | Mylotarg |
|  | glasdegib | Daurismo |
| **H** | histamine dihydrochloride | Ceplene |
| **I** | ibritumomab tiuxetan | Zevalin |
|  | ibrutinib | Imbruvica |
|  | ibuprofen | Pedea |
|  | idecabtagene vicleucel | Abecma |
|  | imatinib | Glivec Imatinib Accord Imatinib Koanaa Imatinib Teva |
|  | inotersen | Tegsedi |
|  | inotuzumab ozogamicin | Besponsa |
|  | interferon beta-1a | Avonex Rebif |
|  | ipilimumab | Yervoy |
|  | irinotecan hydrochloride trihydrate | Onivyde pegylated liposomal (previously known as Onivyde) |
|  | ivosidenib | Tibsovo |
|  | ixazomib | Ninlaro |
| **L** | lenalidomide | Lenalidomide Accord Lenalidomide Krka (previously Lenalidomide Krka d.d. Novo mesto) Lenalidomide Krka d.d. Lenalidomide Krka d.d. Novo mesto (previously Lenalidomide Krka) Lenalidomide Mylan Revlimid |
|  | lenvatinib | Kisplyx Lenvima |
|  | lisocabtagene maraleucel | Breyanzi |
|  | loncastuximab tesirine | Zynlonta |
|  | lutetium (177 Lu) chloride | EndolucinBeta Lumark Lutetium (177Lu) chloride Billev (previously Illuzyce) |
|  | lutetium (177Lu) oxodotreotide | Lutathera |
|  | lutetium (177Lu) vipivotide tetraxetan | Pluvicto |
| **M** | melphalan chloride | Phelinun |
|  | melphalan flufenamide | Pepaxti |
|  | mercaptopurine | Xaluprine (previously Mercaptopurine Nova Laboratories) |
|  | mosunetuzumab | Lunsumio |
|  | mycophenolate mofetil | CellCept Myclausen Mycophenolate mofetil Teva Myfenax |
| **N** | nelarabine | Atriance |
|  | nilotinib | Tasigna |
|  | niraparib | Zejula |
|  | niraparib, abiraterone acetate | Akeega |
|  | nitric oxide | INOmax |
| **O** | obinutuzumab | Gazyvaro |
| **P** | paclitaxel | Abraxane Apealea Pazenir |
|  | palbociclib | Ibrance |
|  | panobinostat | Farydak |
|  | pazopanib | Votrient |
|  | pembrolizumab | Keytruda |
|  | pixantrone dimaleate | Pixuvri |
|  | polatuzumab vedotin | Polivy |
|  | pomalidomide | Imnovid (previously Pomalidomide Celgene) |
|  | ponatinib | Iclusig |
|  | pralsetinib | Gavreto |
| **R** | radium Ra223 dichloride | Xofigo |
|  | ramucirumab | Cyramza |
|  | regorafenib | Stivarga |
|  | rituximab | Blitzima MabThera Rixathon Riximyo Ruxience Truxima |
|  | rucaparib | Rubraca |
|  | ruxolitinib | Jakavi Opzelura |
| **S** | saquinavir | Invirase |
|  | selinexor | Nexpovio |
|  | siltuximab | Sylvant |
|  | sirolimus | Hyftor Rapamune |
|  | sofosbuvir | Sovaldi |
|  | sunitinib | Sunitinib Accord Sutent |
| **T** | tafasitamab | Minjuvi |
|  | tagraxofusp | Elzonris |
|  | talazoparib | Talzenna |
|  | teclistamab | Tecvayli |
|  | tegafur, gimeracil, oteracil | Teysuno |
|  | temsirolimus | Torisel |
|  | thalidomide | Thalidomide BMS (previously Thalidomide Celgene) Thalidomide Lipomed |
|  | thiotepa | Tepadina Thiotepa Riemser |
|  | tisagenlecleucel | Kymriah |
|  | topotecan | Hycamtin Potactasol Topotecan Hospira |
|  | trabectedin | Yondelis |
|  | trastuzumab | Herceptin Herzuma Kanjinti Ogivri Ontruzant Trazimera Zercepac |
|  | trastuzumab deruxtecan | Enhertu |
|  | trastuzumab emtansine | Kadcyla |
|  | tremelimumab | Imjudo Tremelimumab AstraZeneca |
|  | treosulfan | Trecondi |
|  | trifluridine, tipiracil | Lonsurf |
| **V** | venetoclax | Venclyxto |
|  | vinflunine | Javlor |
|  | volanesorsen | Waylivra |
| **Z** | zanubrutinib | Brukinsa |

**D) INNs & TRADE/BRAND NAMES OF DRUGS with ANEMIA, LEUCOPENIA AND THROMBOCYTOPENIA with very common frequency**

| **INN beginning letter** | **INN** | **Trade/Brand names** |
| --- | --- | --- |
| **A** | abemaciclib | Verzenios |
|  | alpelisib | Piqray |
|  | asciminib | Scemblix |
|  | atezolizumab | Tecentriq |
|  | avapritinib | Ayvakyt |
|  | axicabtagene ciloleucel | Yescarta |
|  | azacitidine | Blenrep |
| **B** | belantamab mafodotin | Blincyto |
|  | blinatumomab | Bortezomib Accord Bortezomib Hospira Bortezomib Sun Bortezomib Fresenius Kabi Velcade |
|  | bortezomib | Bosulif |
|  | bosutinib | Tecartus |
|  | Brexucabtagene autoleucel | Busilvex Busulfan Fresenius Kabi |
|  | busulfan | Cabazitaxel Accord Jevtana |
| **C** | cabazitaxel | Capecitabine Accord Capecitabine Medac Capecitabine Teva Ecansya (previously Capecitabine Krka) Xeloda |
|  | capecitabine | Kyprolis |
|  | carfilzomib | Libtayo |
|  | cemiplimab | Carvykti |
|  | ciltacabtagene autoleucel | Mavenclad Litak |
|  | cladribine | Xalkori |
|  | crizotinib | Darzalex |
| **D** | daratumumab | Sprycel |
|  | dasatinib (anhydrous) | Dacogen |
|  | decitabine | Qarziba (previously Dinutuximab beta EUSA and Dinutuximab beta Apeiron) |
|  | dinutuximab beta | Docetaxel Accord Docetaxel Kabi Taxotere |
|  | docetaxel | Caelyx pegylated liposomal Celdoxome pegylated liposomal Myocet liposomal (previously Myocet) Zolsketil pegylated liposomal |
|  | doxorubicin | Imfinzi |
|  | durvalumab | Copiktra |
|  | duvelisib | Inrebic |
| **F** | fedratinib | Faslodex Fulvestrant Mylan |
|  | fulvestrant | Mylotarg |
| **G** | gemtuzumab ozogamicin | Daurismo |
|  | glasdegib | Zevalin |
| **I** | ibritumomab tiuxetan | Abecma |
|  | idecabtagene vicleucel | Glivec Imatinib Accord Imatinib Koanaa Imatinib Teva |
|  | imatinib | Besponsa |
|  | inotuzumab ozogamicin | Avonex Rebif |
|  | interferon beta-1a | Yervoy |
|  | ipilimumab | Onivyde pegylated liposomal (previously known as Onivyde) |
|  | irinotecan hydrochloride trihydrate | Tibsovo |
|  | ivosidenib | Lenalidomide Accord Lenalidomide Krka (previously Lenalidomide Krka d.d. Novo mesto) Lenalidomide Krka d.d. Lenalidomide Krka d.d. Novo mesto (previously Lenalidomide Krka) Lenalidomide Mylan Revlimid |
| **L** | lenalidomide | Kisplyx Lenvima |
|  | lenvatinib | Breyanzi |
|  | lisocabtagene maraleucel | Zynlonta |
|  | loncastuximab tesirine | Zynlonta |
|  | lutetium (177 Lu) chloride | EndolucinBeta Lumark Lutetium (177Lu) chloride Billev (previously Illuzyce) |
|  | lutetium (177Lu) oxodotreotide | Lutathera |
|  | lutetium (177Lu) vipivotide tetraxetan | Pluvicto |
| **M** | melphalan chloride | Phelinun |
|  | melphalan flufenamide | Pepaxti |
|  | mosunetuzumab | Lunsumio |
|  | mycophenolate mofetil | CellCept Myclausen Mycophenolate mofetil Teva Myfenax |
| **N** | nelarabine | Atriance |
|  | niraparib | Zejula |
|  | niraparib, abiraterone acetate | Akeega |
| **O** | obinutuzumab | Gazyvaro |
| **P** | paclitaxel | Abraxane Apealea Pazenir |
|  | palbociclib | Ibrance |
|  | panobinostat | Farydak |
|  | pembrolizumab | Keytruda |
|  | pixantrone dimaleate | Pixuvri |
|  | polatuzumab vedotin | Polivy |
|  | pomalidomide | Imnovid (previously Pomalidomide Celgene) |
|  | pralsetinib | Gavreto |
| **R** | ramucirumab | Cyramza |
|  | rucaparib | Rubraca |
|  | ruxolitinib | Jakavi Opzelura |
| **S** | selinexor | Nexpovio |
|  | sirolimus | Hyftor Rapamune |
|  | sofosbuvir | Sovaldi |
|  | sunitinib | Sunitinib Accord Sutent |
| **T** | tafasitamab | Minjuvi |
|  | talazoparib | Talzenna |
|  | teclistamab | Tecvayli |
|  | tegafur, gimeracil, oteracil | Teysuno |
|  | temsirolimus | Torisel |
|  | thalidomide | Thalidomide BMS (previously Thalidomide Celgene) Thalidomide Lipomed |
|  | thiotepa | Tepadina Thiotepa Riemser |
|  | tisagenlecleucel | Kymriah |
|  | topotecan | Hycamtin Potactasol Topotecan Hospira |
|  | trabectedin | Yondelis |
|  | trastuzumab | Herceptin Herzuma Kanjinti Ogivri Ontruzant Trazimera Zercepac |
|  | trastuzumab deruxtecan | Enhertu |
|  | tremelimumab | Imjudo Tremelimumab AstraZeneca |
|  | treosulfan | Trecondi |
|  | trifluridine, tipiracil | Lonsurf |
| **V** | venetoclax | Venclyxto |
|  | vinflunine | Javlor |
| **Z** | zanubrutinib | Brukinsa |
